# Collodion in Erysipelas

**Published:** 1852-07

**Authors:** M. Latour


From Revue Medicale, 1851, p. 462.

*On the Application of Collodion in Erysipelas.* By. M. LATOUR.

M. LATOUR, believing that erysipelas is best treated by the application of an impermeable covering to the skin, has experimented with a very great variety of substances, and pronounces collodion to be the best. Spread out on the surface, however, it sometimes so compresses the skin as to cause pain and irritation—phlyctenæ also forming when it cracks. The following combination, however, remedies these inconveniences completely:—*Collodion*, 30 *grammes; Venice turpentine*, 15 *decigrammes; castor oil*, 5 *decigrammes;* to be mixed by shaking. It is easily detached by a linseed poultice. The following is also used with great success, rarely irritating the skin:—*Collodion*, 30 *parts; old castor oil*, 2 *parts.*

